# Differences in the attitudes towards resuscitation of extremely premature infants between neonatologists and obstetricians: a survey study in China

**DOI:** 10.3389/fped.2023.1308770

**Published:** 2023-12-13

**Authors:** Dan Wang, Li Li, Bo-Wen Ming, Chun-Quan Ou, Tao Han, Jingke Cao, Wenyu Xie, Changgen Liu, Zhichun Feng, Qiuping Li

**Affiliations:** ^1^ Department of Newborn Care Center, Senior Department of Pediatrics, The Seventh Medical Center of PLA General Hospital, Beijing, China; ^2^ The Second School of Clinical Medicine, Southern Medical University, Guangzhou, China; ^3^ National Engineering Laboratory for Birth Defects Prevention and Control of Key Technology, Beijing, China; ^4^ Beijing Key Laboratory of Pediatric Organ Failure, Beijing, China; ^5^ State Key Laboratory of Organ Failure Research, Department of Biostatistics, Guangdong Provincial Key Laboratory of Tropical Disease Research, School of Public Health, Southern Medical University, Guangzhou, China

**Keywords:** attitudes, extremely preterm infants, neonatologists, obstetricians, resuscitation

## Abstract

**Objectives:**

Neonatologists and obstetricians are crucial decision-makers regarding the resuscitation of extremely preterm infants (EPIs). However, there is a scarcity of research regarding the differing perspectives on EPI resuscitation between these medical professionals. We aim to determine the differences and influential factors of their attitudes towards EPIs resuscitation in China.

**Methods:**

This cross-sectional study was conducted in public hospitals of 31 provinces in Chinese mainland from June to July 2021. Influential factors of binary variables and those of ordinal variables were analyzed by modified Poisson regression models and multinomial logistic regression models due to the invalid parallel line assumption of ordinal logistic regression models.

**Results:**

A total of 832 neonatologists and 1,478 obstetricians who were deputy chief physicians or chief physicians participated. Compared with obstetricians, neonatologists delivered a larger proportion of infants of <28-week gestational age (87.74% vs. 84.91%) and were inclined to think it inappropriate to use 28 weeks as the cutoff of gestational age for providing full care to premature infants [63.34% vs. 31.60%, adjusted prevalence ratio = 1.61 (95% CI: 1.46–1.77)], and to suggest smaller cutoffs of gestational age and birth weight for providing EPIs resuscitation. Notably, 46.49% of the neonatologists and 19.01% of the obstetricians believed infants ≤24 weeks' gestation should receive resuscitation.

**Conclusions:**

In China, notable disparities exist in attitudes of neonatologists and obstetricians towards resuscitating EPIs. Strengthening collaboration between these two groups and revising the pertinent guidelines as soon as possible would be instrumental in elevating the resuscitation rate of EPIs.

## Introduction

Over the past decades, significant progress has been made in the treatment of extremely premature infants (EPIs) due to advancements in medical technologies and improved collaboration between obstetricians and neonatologists (1). EPIs are at high risk of mortality and morbidity due to their extremely immature organ and tissue development (2, 3). The mortality and risk of sequelae increase with decreasing gestational age (4). Consequently, there has been a long-standing medical and ethical debate on the resuscitation threshold for providing full care to premature infants (5). Resuscitation practices and survival rates for EPIs vary widely across countries and regions, hospitals, and practitioners (6). Developed countries generally have a positive attitude towards resuscitation, with over 93% of premature infants at 24 weeks receiving active treatment (7). In the United States of America, proactive neonatal resuscitation starting at 22 weeks of gestation is supported (8). However, most developing countries have relatively low rates of resuscitation acceptance and survival for EPI (9). In China, the current cutoff for providing full care to premature infants is 28 weeks of gestational age (10). In most cases, EPIs receive minimal resuscitation in the delivery room and only a small proportion of them receive active treatment in the neonatal intensive care unit (NICU) (11). The high rate of resuscitation withdrawal for EPIs may be partly attributed to the conservative attitudes of obstetricians. Parents mostly rely on information about the prognosis of their EPIs from obstetricians and neonatologists.

In China, obstetricians are the primary communicators with parents in the delivery room, while neonatologists are not always involved in prenatal consultations (11). According to our previous study, most Chinese obstetricians believe that providing full care to premature infants should be limited to those born at 28 weeks of gestation (12). However, neonatologists hold a more positive attitude towards treating EPIs, suggesting a divergence in opinions between obstetricians and neonatologists. Differences in positions, responsibilities, and perceptions regarding EPI care and prognosis between obstetricians and neonatologists may influence their attitudes towards EPI resuscitation. A study conducted in England revealed differing attitudes between obstetricians and neonatologists when counseling parents facing preterm birth on the treatment of EPIs (13).

By shedding light on the divergent attitudes towards EPI resuscitation and the underlying reasons among obstetricians and neonatologists in China, we aim to enhance collaboration between the two specialties and subsequently improve the survival rates of EPIs. To this end, we conducted a large-scale national survey on the attitudes towards EPI resuscitation among neonatologists and obstetricians in China, intending to improve EPI resuscitation and make some revisions on the related guidelines for EPIs.

## Materials and methods

### Survey design

This cross-sectional study was conducted in neonatologists and obstetricians registered in public hospitals of 31 provinces (including autonomous regions or municipalities) in the Chinese mainland between June and July 2021. Questionnaires were distributed via the online survey platform “Wenjuanxing” in Wechat groups composed of neonatal and obstetric experts in China, and then forwarded to neonatologists and obstetricians in their provinces. Here, we focused on the attitudes of deputy chief physicians and chief physicians, since these doctors are more experienced than others. Finally, a total of 832 neonatologists and 1,478 obstetricians who were deputy chief physicians or chief physicians participated in this study.

According to the different perspectives of neonatologists and obstetricians, two questionnaire forms were designed after repeated discussions and revisions by hospital management experts and senior neonatal and obstetric experts. The corresponding contents of the two questionnaire forms were consistent. Obstetrician's questionnaire survey methods including questionnaire distribution and data collection have been partial reported in our previous study, but in this study we explored the data of deputy chief physicians and chief physicians more deeply (12).

This study was approved by the research ethics board of the Seventh Medical Center of PLA General Hospital (No. 2021-104). Written informed consent has been obtained from all participants involved in this study. The manuscript has been carefully reviewed to ensure that no natural and identifiable information, such as names or hospital numbers, is included.

### Data collection

Data collected in this study included the demographics and professional experiences of the participating doctors, characteristics of their working hospitals, their attitudes towards the relative importance of ten potentially influential factors concerning the decision-making of EPI resuscitation, requests of the parents or legal guardians of EPIs for giving resuscitation, personal experiences of being in a dilemma about the decision on sending EPIs to the NICU for treatment and the reasons, the attitude towards whether it was appropriate to use 28 weeks as the cutoff for providing full care to premature infants, and the lowest gestational age and birth weight of EPIs that should receive resuscitation.

### Statistical analysis

The continuous variable of respondents’ age was summarized as the mean and standard deviation (SD). Categorical and ordinal variables are were expressed as numbers and corresponding percentages. The mean age between neonatologists and obstetricians was compared by using the *t*' test, for the equal variance assumption was invalid. Differences in ordinal variables were assessed by Mann–Whitney *U-*tests, and categorical variables were compared by *χ*^2^ tests.

In addition, we compared the attitudes regarding whether it was inappropriate to use 28 weeks as the cutoff for providing full care to premature infants and the lowest gestational age and birth weight of EPIs that should receive resuscitation between neonatologists and obstetricians, without and with adjustment of the demographics, the working hospitals of the participating doctors and their professional experiences. Specially, we firstly analyzed binary variables (i.e., whether it was inappropriate to use 28 weeks as the cutoff for providing full care to premature infants, whether resuscitation should be given to EPIs no matter how light they were) using modified Poisson regression models without any adjustment (Model 1), since these outcomes were not uncommon and the odds ratios (ORs) obtained from logistic regression cannot approximate prevalence ratios (PRs) in this situation (14, 15). Subsequently, we fitted Model 2 which accounted for demographic variables, and then Model 3 which further included the characteristics of the hospitals concerned and the professional experiences of the participating doctors. In addition, the differences in the lowest gestational age and birth weight of EPIs that should receive resuscitation were assessed using multinomial logistic regression models, for the parallel line assumption of the ordinal logistic regression was invalid. The models were constructed to evaluate the ORs of the attitudes between the neonatologists and obstetricians. Furthermore, we examined the influential factors of these attitudes in neonatologists and obstetricians separately to determine the common influential factors for the two populations.

We did not impute missing data. Two-sided *P *< 0.05 was considered statistically significant. All analyses were conducted using R software version 4.1.2 (R Foundation for Statistical Computing).

## Results

### Characteristics of the participants

The included neonatologists were on average older than obstetricians (48.80 vs*.* 47.30 years). Approximate 95% of the obstetricians were females vs. 68.87% for the neonatologists. The percentages of married doctors and those having children in neonatologists were higher than those in the obstetricians (Table 1). The proportion of the neonatologists working in general hospitals was less than that of the obstetricians (57.57% vs. 73.21%). More neonatologists than obstetricians worked in tertiary hospitals (84.50% vs. 59.95%). Largely speaking, a greater number of EPIs born in the departments where the neonatologists worked. The number of neonatologists who were chief physicians was larger than that of obstetricians with the same professional title (59.86% vs. 37.69%). Compared with the obstetricians, more neonatologists had the experience of delivering infants younger than 28 weeks of gestational age (87.74% vs. 84.91%).

**Table 1:** Characteristics of the study participants.

Characteristics^a^	All participants (*n* = 2,310)	Neonatologists (*n* = 832)	Obstetricians (*n* = 1,478)^b^	*P*
Demographic				
Age, years	47.84 (6.36)	48.80 (6.05)	47.30 (6.47)	<0.001
Sex – female	1,983 (85.84)	573 (68.87)	1,410 (95.40)	<0.001
Han Chinese	2,071 (89.65)	757 (90.99)	1,314 (88.90)	0.115
Marital status				0.582
Married	2,253 (97.53)	815 (97.96)	1,438 (97.29)	
Unmarried	15 (0.65)	5 (0.60)	10 (0.68)	
Others	42 (1.82)	12 (1.44)	30 (2.03)	
Having children	2,268 (98.18)	814 (97.84)	1,454 (98.38)	0.351
Region				<0.001
West of China	782 (33.85)	333 (40.02)	449 (30.38)	
Center of China	598 (25.89)	226 (27.16)	372 (25.17)	
East of China	930 (40.26)	273 (32.81)	657 (44.45)	
Characteristic of working hospital				
Hospital category – general hospital	1,561 (67.58)	479 (57.57)	1,082 (73.21)	<0.001
Hospital level – tertiary hospital	1,589 (68.79)	703 (84.50)	886 (59.95)	<0.001
Annual number of extremely premature infants delivered in the department				<0.001
<10	1,175 (50.87)	330 (39.66)	845 (57.17)	
10–29	522 (22.60)	212 (25.48)	310 (20.97)	
30–50	218 (9.44)	110 (13.22)	108 (7.31)	
>50	395 (17.10)	180 (21.63)	215 (14.55)	
Professional characteristics				
Job title – chief physician	1,055 (45.67)	498 (59.86)	557 (37.69)	<0.001
Having delivered infants at <28 weeks gestation	1,985 (85.93)	730 (87.74)	1,255 (84.91)	0.070

^a^ The continuous variable of age was expressed as mean (standard deviation), while categorical and ordinal variables were summarized as counts and percentages. The *t*’ test was used to compare the means of age between neonatologists and obstetricians. *χ*^2^ tests were applied to categorical variables, while annual number of extremely premature infants born in the department, and job title were compared with Mann–Whitney *U*-tests.

^b^ The sample size for age was 1,473.

### Relative importance of factors affecting decision making of EPI resuscitation

**Figure 1:**
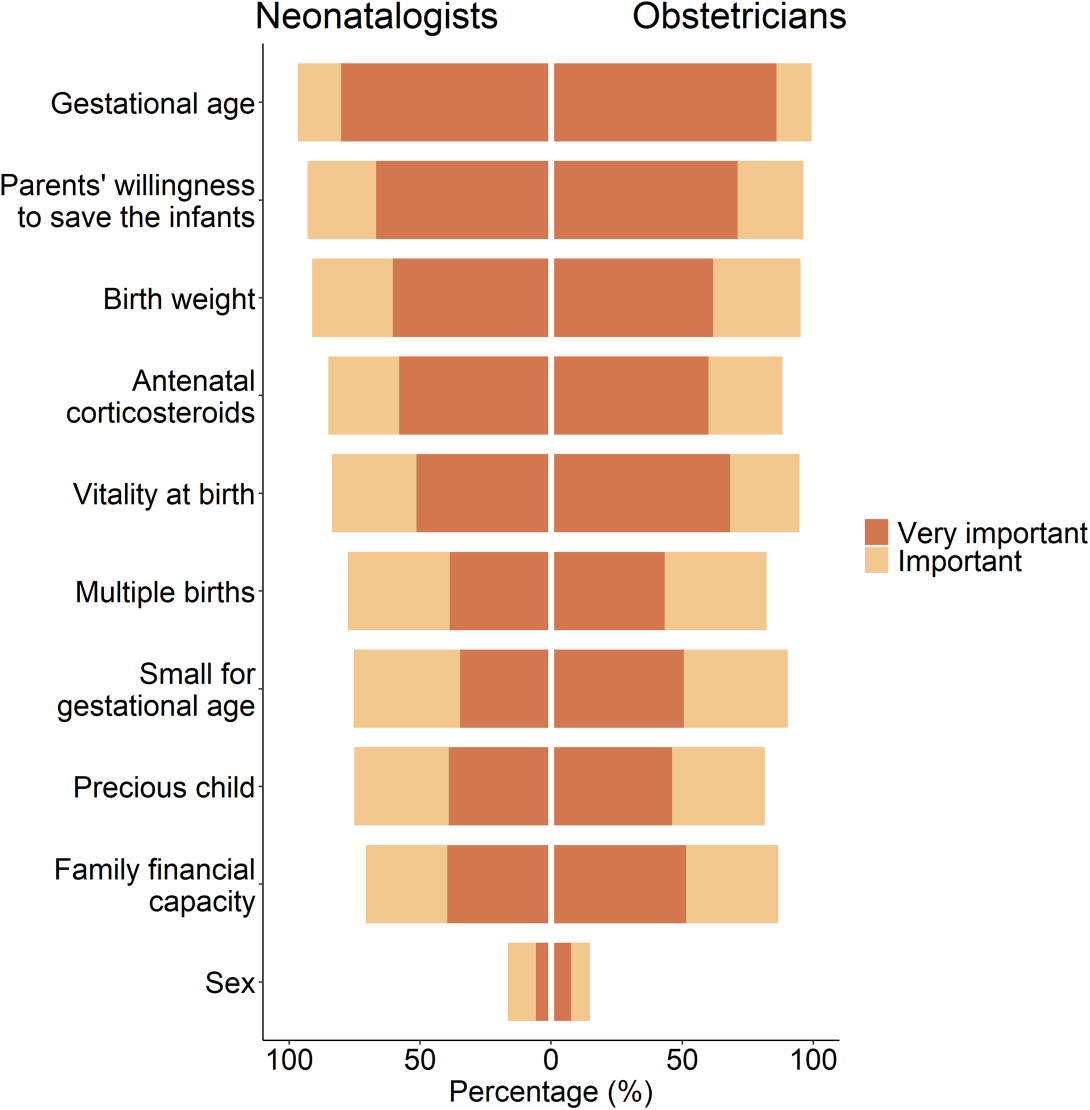
Attitudes towards the relative importance of factors affecting resuscitation decision-making for extreme preterm infants.

As for the relative importance of factors influencing the decision-making for EPI resuscitation, over 60% of the neonatologists and obstetricians thought that gestational age, parents’ willingness to save the infants, and birth weight were very important (Figure 1 and Supplementary Table S1).

### Attitudes and experiences regarding EPI resuscitation withdrawal

More than half of the neonatologists and obstetricians reckoned that factual communication was needed when the family members requested for giving up EPI resuscitation when there was a satisfactory birth condition and high possibility of survival (Table 2). Over 70% of the neonatologists and obstetricians thought that parents should make the final decision on whether to save their EPIs, and the disparity between the neonatologists and obstetricians was statistically significant (*P *< 0.001). Compared with the obstetricians, a higher proportion of the neonatologists experienced the dilemma of whether to send EPIs to the NICU for treatment (72.48% vs. 56.36%). The main reasons for this discrepancy were also different. 50.91% of the neonatologists were due to uncertainty about the prognosis of treatment, and 46.70% of the obstetricians worried about poor prognosis and family disputes.

**Table 2:** Comparisons of the attitudes and experiences regarding withdrawing EPI resuscitation between neonatologists and obstetricians.

Variables^a^	Neonatologists (*n* = 832)^b^	Obstetricians (*n* = 1,478)^c^	*P*
Family members requested for giving up EPI resuscitation when there was a satisfactory birth condition and high possibility of survival			
Actively persuade the parents to treat EPIs	347 (41.71)	634 (42.90)	0.909
Factual communication, depends on parents	474 (56.97)	801 (54.19)	
Do as the parents wish	11 (1.32)	43 (2.91)	
The one who should make the final decision on whether to save EPIs			<0.001
Parents	615 (73.92)	1,042 (70.50)	
Neonatologists	114 (13.70)	345 (23.34)	
Obstetricians	0 (0.00)	22 (1.49)	
Ethics committee	55 (6.61)	39 (2.64)	
Others	48 (5.77)	30 (2.03)	
Being in a dilemma about the decision on sending EPIs to NICU for treatment	603 (72.48)	833 (56.36)	<0.001
Reason for being in a dilemma			<0.001
Uncertain about the prognosis of treatment	307 (50.91)	165 (19.81)	
Worrying about poor prognosis and family disputes	193 (32.01)	389 (46.70)	
Worrying that the family cannot afford it	73 (12.11)	199 (23.89)	
Others	30 (4.98)	80 (9.60)	
You or your colleague have experienced medical disputes	278 (33.41)	560 (37.89)	0.032

EPIs, extreme preterm infants; NICU, newborn intensive care unit.

^a^ Variables were summarized as counts and percentages. *χ*^2^ tests were applied to categorical variables, while the attitudes towards family members requested for giving up EPI resuscitation when there was a satisfactory birth condition and high possibility of survival was compared with Mann–Whitney *U-*tests.

^b^ The sample size for reason of being in a dilemma was 603.

^c^ The sample size for reason of being in a dilemma was 833.

### Attitudes towards the current threshold for providing full care to premature infants

**Figure 2:**
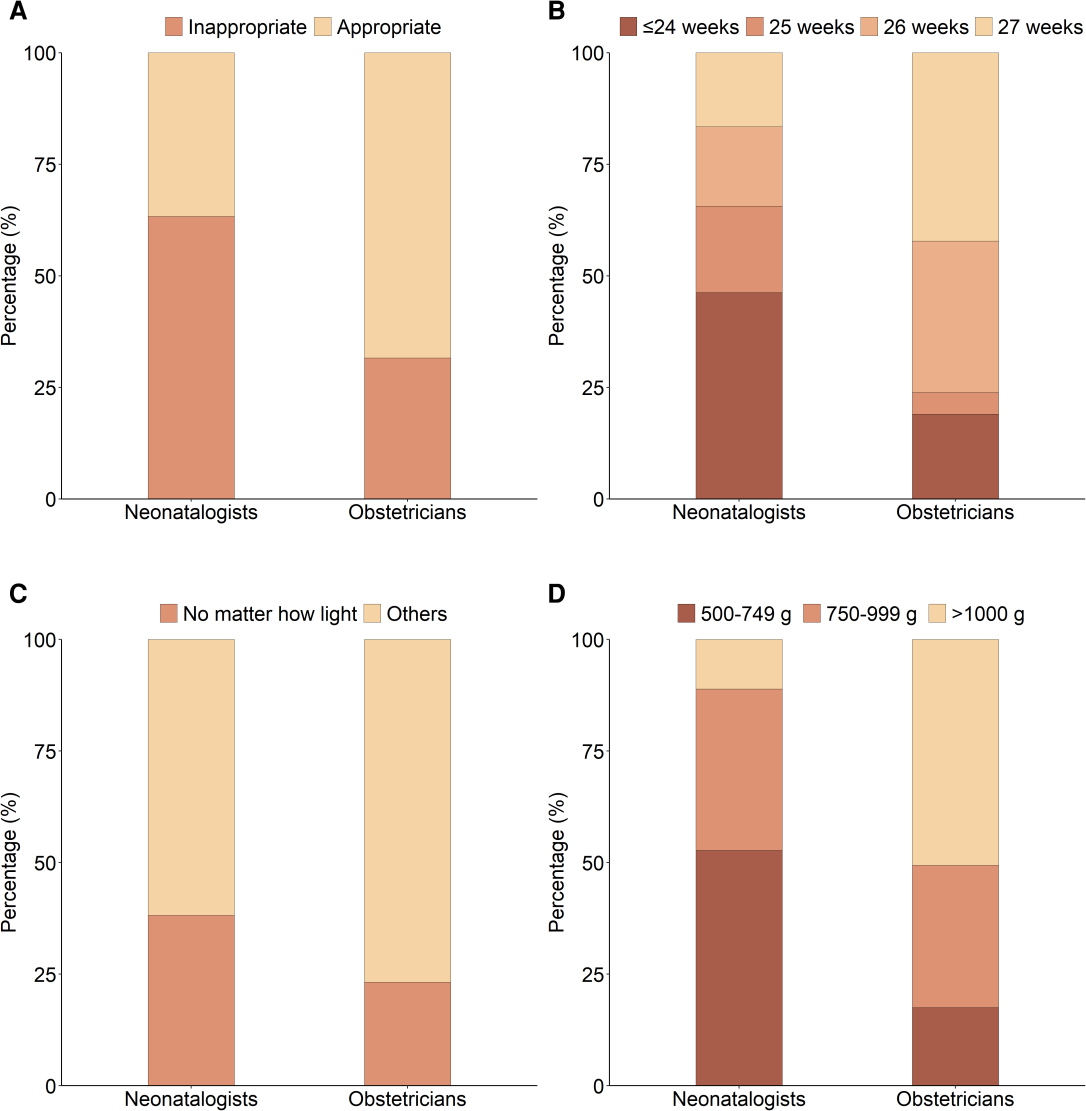
Attitudes towards resuscitation of extremely premature infants. (**A**) Whether it is appropriate to use 28 weeks as the cutoff for providing full care to premature infants; (**B**) The lowest gestational age of EPIs that should receive resuscitation; (**C**) Should EPIs receive resuscitation no matter how light they are; (**D**) The lowest birth weight of EPIs that should receive resuscitation.

Compared with the obstetricians, more neonatologists thought it inappropriate to use 28 weeks as the cutoff for providing full care to premature infants (63.34% vs. 31.60%; Figure 2 and Supplementary Table S2), even after adjusting for demographic variables, characteristics of the hospitals concerned and professional experiences of the participating doctors [PR = 1.61 (95% CI: 1.46, 1.77); Table 3 and Supplementary Table S3]. Both neonatologists and obstetricians who had the experience of delivering infants with gestational age <28 weeks tended to think that 28 weeks was not an appropriate cutoff for providing full care to premature infants (Supplementary Table S4).

**Table 3:** Comparisons of the attitudes towards resuscitation of extremely premature infants between neonatologists and obstetricians.

Variables	Model 1		Model 2		Model 3	
	PR (95% CI)^a^	*P*	PR (95% CI)	*P*	PR (95% CI)	*P*
28 weeks as the cutoff for providing full care to premature infants is inappropriate^b^	2.00 (1.83, 2.20)	<.001	1.86 (1.69, 2.04)	<.001	1.61 (1.46, 1.77)	<.001
The lowest gestational age of EPIs receiving resuscitation (weeks)^c^						
27	Reference		Reference		Reference	
26 vs. 27	1.34 (1.03, 1.73)	0.029	1.21 (0.92, 1.61)	0.180	1.07 (0.80, 1.44)	0.651
25 vs. 27	10.11 (7.24, 14.11)	<.001	8.54 (5.96, 12.24)	<.001	6.78 (4.64, 9.91)	<.001
≤24 vs. 27	6.20 (4.87, 7.88)	<.001	5.46 (4.20, 7.09)	<.001	4.49 (3.39, 5.94)	<.001
EPIs should receive resuscitation no matter how light they are^a^	1.65 (1.45, 1.87)	<.001	1.73 (1.51, 1.98)	<.001	1.77 (1.53, 2.04)	<.001
The lowest birth weight of EPIs receiving resuscitation (g)^b^						
>1,000	Reference		Reference		Reference	
750–999 vs. >1,000	5.17 (3.74, 7.16)	<.001	5.50 (3.83, 7.88)	<.001	5.32 (3.62, 7.82)	<.001
500–749 vs. >1,000	13.72 (9.88, 19.04)	<.001	14.10 (9.76, 20.37)	<.001	13.96 (9.33, 20.89)	<.001

EPIs, extreme preterm infants; 95% CI, 95% confidence interval.

^a^ PR means the prevalence ratio or odds ratio comparing neonatologists with obstetricians; Model 1: adjusted for nothing; Model 2: adjusted for demographic characteristics (i.e. age, sex, ethnicity, marital status, having kids or not, and region); Model 3: adjusted for all of the variables listed in Table 1.

^b^ Modified Poisson regression models were used to evaluate the difference between neonatologists and obstetricians. The estimator was prevalence ratio.

^c^ Multinomial logistic regression models were applied to assess the difference between neonatologists and obstetricians. The estimator was odds ratio.

### Attitudes regarding the lowest gestational age of EPIs receiving resuscitation

Compared with the obstetricians, the neonatologists tended to suggest an even lower limit for the lowest gestational age of EPIs receiving resuscitation (Table 3 and Supplementary Table S5). Notably, 46.49% of the neonatologists proposed that the lowest gestational age could be ≤24 weeks, vs. 19.01% for the obstetricians (Figure 2). Region, the characteristics of working hospitals, and the professional experiences of the participating doctors except for job title were associated with the attitudes towards the lowest gestational age of EPIs receiving resuscitation for both neonatologists and obstetricians (Supplementary Tables S6,S7).

### Attitudes towards the lowest birth weight of EPIs receiving resuscitation

Compared with the obstetricians, the neonatologists were more likely to believe that EPIs should receive resuscitation no matter how light they were [38.22% vs. 23.21%, adjusted for covariates: PR = 1.77 (95% CI: 1.53, 2.04); Figure 2, Table 3 and Supplementary Table S8]. It seemed that there was no common factor of the attitudes towards whether EPIs should receive resuscitation no matter how light they were for both neonatologists and obstetricians (Supplementary Table S9).

In addition, compared with the obstetricians, more neonatologists thought that the lowest birth weight should be lighter for EPIs who should receive resuscitation (Figure 2, Table 3, and Supplementary Table S10). Both neonatologists and obstetricians who worked in hospitals in the east of China, tertiary hospitals, the department with ≥10 premature infants annually, and those with the practice of delivering infants with gestational age <28 weeks tended to suggest a smaller lowest birth weight (Supplementary Table S11).

## Discussion

The treatment of EPIs necessitates collaborative efforts between obstetricians and neonatologists (16). If both parties maintain a consistently positive attitude towards EPI resuscitation, it will enhance the treatment opportunities for these infants. Conversely, a less optimistic attitude from either of the two parties would result in missed treatment opportunities for EPIs. Given the low EPI resuscitation and survival rates in China, an investigation into differing attitudes between obstetricians and neonatologists toward EPI resuscitation is warranted. To the best of our knowledge, this is the inaugural nationwide cross-sectional study on differing attitudes towards EPI resuscitation between obstetricians and neonatologists in China. As the final clinical decisions are typically made by deputy chief physicians and chief physicians in China, and parents are more likely to trust their judgment on whether to resuscitate their EPIs, we specifically focused on their attitudes. Our findings revealed that obstetricians generally exhibited a more conservative attitude compared with neonatologists.

Conversely, resuscitation practices for EPIs in developing countries have not been as aggressive as those in developed countries. In countries such as South Africa, Lebanon, and Malaysia, infant resuscitation is generally not considered at gestational ages ≤25 weeks (17–19). As for Mongolia, resuscitation is typically performed for premature infants at gestational ages ranging from 28 to 31 weeks, which represents the earliest gestational age for resuscitation (20). China, being a developing country, shares a similar situation. It was found in our study that only 19.01% of obstetricians acknowledged ≤24 weeks as the lowest gestational age for EPI resuscitation.

Our study also revealed that neonatologists exhibited a more positive attitude compared with obstetricians regarding the lowest gestational age at which EPIs should receive resuscitation and the current cutoff for providing comprehensive care to premature infants. These findings indicate significant differences in their attitudes towards EPI resuscitation in China. This finding is consistent with a previous study conducted in Brazil, which highlighted disagreements among obstetricians regarding the proactive management of EPIs (21). In Brazil, there was also a communication gap between obstetricians and neonatologists, with obstetricians tending to underestimate the viability of EPIs. Moreover, the utilization of antenatal steroids and Cesarean section was significantly lower in Brazil, compared with the US National Institute of Child Health and Human Development Neonatal Research Network (21, 22). Similarly, in Mongolia, obstetricians were more inclined to withdraw neonatal resuscitation compared with neonatologists (23).

Two factors may account for the discrepancies in attitudes toward EPI resuscitation between neonatologists and obstetricians in China. Firstly, there are distinct characteristics associated with neonatologists and obstetricians. Neonatologists typically work in high-level hospitals and possess more experience in EPI resuscitation. Over time, the number of NICUs in China has significantly increased, particularly in large tertiary hospitals situated in major cities (24). A study indicated that physicians working in NICUs were more inclined to opt for a lower gestational age threshold compared with other healthcare professionals (25). Moreover, many parents of EPIs prefer to deliver their babies to large tertiary hospitals or transfer them to better-equipped facilities after initial delivery in local hospitals, mirroring a trend observed in developed countries (26). Secondly, there is currently a lack of consensus guidelines for both neonatologists and obstetricians. The capacity for EPI resuscitation has rapidly improved in recent years with the notable socio-economic development in China. Survival rates and prognosis for EPI treatment in neonatology departments, particularly those in Class A tertiary hospitals, have shown continuous advancement like other countries (27). However, obstetric guidelines have not kept pace with these advancements. In national obstetrical guidelines, the current definition of the perinatal period in China is from 28 weeks of pregnancy to seven days after birth, with the gestational age of 28 weeks suggested as the starting point for active treatment of premature infants (10). These factors have a great impact on the attitudes of obstetricians towards EPI resuscitation (28).

Currently, obstetricians in China continue to serve as the primary source of prenatal counseling for EPI resuscitation, often without the involvement of neonatologists during these consultations, which may cause an area of controversy and raises many ethical and legal issues (29–31). As a result of the divergent attitudes towards EPI resuscitation between neonatologists and obstetricians, it has become common practice to withdraw resuscitation efforts for EPIs in the delivery room (11). A survey in China indicated that the rate of withholding resuscitation for EPIs between 24 and 27 weeks of gestation in the delivery room was alarmingly high as 73%, suggesting that a significant number of potentially viable EPIs did not have the opportunity to receive treatment in NICUs (11, 24, 32). This issue has also contributed to doctor-patient disputes in China. Our study discovered that nearly one-third of participants, or their colleagues, had encountered medical disputes connected to EPI resuscitation. Thus, it is crucial to urgently revise the current obstetric guidelines and reassess the concept of perinatal periods, and at the same time, the collaboration between obstetricians and neonatologists should be strengthened. We believe that both the proportion and quality of EPI resuscitation can be improved after implementing these changes (33).

Although there are notable differences in attitudes towards EPI resuscitation between neonatologists and obstetricians, they share a common understanding of the key factors influencing resuscitation decision-making for EPIs. Both groups consider gestational age, parents’ willingness, and birth weight as the primary factors influencing the decision on EPI resuscitation. This aligns with the findings from international studies as well. For instance, a previous study identified gestational age as the primary factor influencing resuscitation decisions (34). Guidelines for EPI resuscitation often highlight gestational age and birth weight as major considerations (35). Additionally, gestational age and birth weight have been individually used as predictors of survival rates or the rates of survival without significant impairment (36). Moreover, parental opinions are also regarded as one of the most influential factors in the final decision-making process (37–39). When EPIs exhibit a high likelihood of gaining a favorable prognosis, obstetricians and neonatologists tend to proactively engage in communication and have consonance to persuade parents to consent to resuscitation and treatment for their infants. However, both neonatologists and obstetricians face challenges when it comes to resuscitation decisions, particularly when considering potential poor prognoses and conflicts (40).

Three limitations of this study should be mentioned. First, the participating neonatologists and obstetricians were not selected randomly. However, the included doctors came from all 31 provinces in mainland China and the sample size was comparatively large. Second, the findings of our study may not be generalized to all of the countries globally. However, our results would have an important implication on how to improve the survival rate of EPIs in China as well as the regions which have similar characteristics as China. Thirdly, we did not assess how much guidelines impact physicians, especially obstetricians' attitudes.

In conclusion, obstetricians generally exhibit a more conservative attitude compared with neonatologists towards EPI resuscitation. Meanwhile, they share a common understanding of the key factors influencing resuscitation decision-making for EPIs. It is imperative to enhance perinatal collaboration to improve the resuscitation rate of EPIs. Furthermore, it is necessary to prioritize the reinforcement of ethical and legal frameworks in this regard.

## Data Availability

The original contributions presented in the study are included in the article/**Supplementary Material**, further inquiries can be directed to the corresponding authors.
