# Supplementary material for: Differences in the attitudes towards resuscitation of extremely premature infants between neonatologists and obstetricians: a survey study in China
Source: Front Pediatr. 2023 Dec 13;11:1308770. doi: 10.3389/fped.2023.1308770 (PMC10751309; doi:10.3389/fped.2023.1308770)
Supplement: Supplementary file 1 [file Table1.docx]

**Table S1** Attitudes towards the relative importance of potentially influential factors of resuscitation decision-making.

| Factors | Neonatologists  (n=832) | Obstetricians (n=1478) | *P* |
| --- | --- | --- | --- |
| Gestational age |  |  | 0.001 |
| Very important | 666 (80.05) | 1271 (85.99) |  |
| Important | 138 (16.59) | 197 (13.33) |  |
| Sometimes important | 16 (1.92) | 7 (0.47) |  |
| Less important | 7 (0.84) | 3 (0.20) |  |
| Unimportant | 5 (0.60) | 0 (0.00) |  |
| Birth weight |  |  | 0.172 |
| Very important | 502 (60.34) | 912 (61.71) |  |
| Important | 257 (30.89) | 494 (33.42) |  |
| Sometimes important | 38 (4.57) | 59 (3.99) |  |
| Less important | 21 (2.52) | 12 (0.81) |  |
| Unimportant | 14 (1.68) | 1 (0.07) |  |
| Sex |  |  | 0.037 |
| Very important | 49 (5.89) | 112 (7.58) |  |
| Important | 89 (10.70) | 107 (7.24) |  |
| Sometimes important | 92 (11.06) | 136 (9.20) |  |
| Less important | 166 (19.95) | 277 (18.74) |  |
| Unimportant | 436 (52.40) | 846 (57.24) |  |
| Vitality at birth |  |  | <0.001 |
| Very important | 427 (51.32) | 1009 (68.27) |  |
| Important | 269 (32.33) | 391 (26.45) |  |
| Sometimes important | 68 (8.17) | 50 (3.38) |  |
| Less important | 31 (3.73) | 15 (1.01) |  |
| Unimportant | 37 (4.45) | 13 (0.88) |  |
| Prenatal hormones |  |  | 0.144 |
| Very important | 482 (57.93) | 888 (60.08) |  |
| Important | 225 (27.04) | 417 (28.21) |  |
| Sometimes important | 52 (6.25) | 69 (4.67) |  |
| Less important | 42 (5.05) | 63 (4.26) |  |
| Unimportant | 31 (3.73) | 41 (2.77) |  |
| Multiple births |  |  | 0.008 |
| Very important | 322 (38.70) | 639 (43.23) |  |
| Important | 323 (38.82) | 575 (38.90) |  |
| Sometimes important | 74 (8.89) | 98 (6.63) |  |
| Less important | 64 (7.69) | 92 (6.22) |  |
| Unimportant | 49 (5.89) | 74 (5.01) |  |
| Small for gestational age |  |  | <0.001 |
| Very important | 289 (34.74) | 747 (50.54) |  |
| Important | 337 (40.50) | 587 (39.72) |  |
| Sometimes important | 93 (11.18) | 79 (5.35) |  |
| Less important | 64 (7.69) | 40 (2.71) |  |
| Unimportant | 49 (5.89) | 25 (1.69) |  |
| Family financial capability |  |  | <0.001 |
| Very important | 330 (39.66) | 762 (51.56) |  |
| Important | 258 (31.01) | 519 (35.12) |  |
| Sometimes important | 130 (15.62) | 128 (8.66) |  |
| Less important | 61 (7.33) | 31 (2.10) |  |
| Unimportant | 53 (6.37) | 38 (2.57) |  |

*Note*. The attitudes between neonatologists and obstetricians were compared with Mann-Whitney U tests.

Abbreviation: EPIs, extreme preterm infants.

**Table S1** Attitudes towards the relative importance of potentially influential factors of resuscitation decision-making. *continued*

| Factors | Neonatologists  (n=832) | Obstetricians (n=1478) | *P* |
| --- | --- | --- | --- |
| Precious child |  |  | <0.001 |
| Very important | 325 (39.06) | 681 (46.08) |  |
| Important | 300 (36.06) | 524 (35.45) |  |
| Sometimes important | 106 (12.74) | 129 (8.73) |  |
| Less important | 50 (6.01) | 73 (4.94) |  |
| Unimportant | 51 (6.13) | 71 (4.80) |  |
| Parents’ willingness to save the infants |  |  | 0.010 |
| Very important | 555 (66.71) | 1051 (71.11) |  |
| Important | 218 (26.20) | 371 (25.10) |  |
| Sometimes important | 35 (4.21) | 36 (2.44) |  |
| Less important | 7 (0.84) | 10 (0.68) |  |
| Unimportant | 17 (2.04) | 10 (0.68) |  |

*Note*. The attitudes between neonatologists and obstetricians were compared with Mann-Whitney U tests.

Abbreviation: EPIs, extreme preterm infants.

**Table S2** Attitudes towards resuscitation of extremely premature infants.

| Attitude | Neonatologists (n=832) | Obstetricians (n=1478) | *P* |
| --- | --- | --- | --- |
| Whether it is appropriate to use 28 weeks as the cutoff for providing full care to premature infants |  |  | <0.001 |
| Inappropriate | 527 (63.34) | 467 (31.60) |  |
| Appropriate | 305 (36.66) | 1011 (68.40) |  |
| The lowest gestational age of EPIs that should receive resuscitation |  |  | <0.001 |
| ≤24 weeks | 385 (46.27) | 281 (19.01) |  |
| 25 weeks | 161 (19.35) | 72 (4.87) |  |
| 26 weeks | 148 (17.79) | 501 (33.90) |  |
| 27 weeks | 138 (16.59) | 624 (42.22) |  |
| Should EPIs receive resuscitation no matter how light they are |  |  |  |
| No matter how light | 318 (38.22) | 343 (23.21) | <0.001 |
| Others | 514 (61.78) | 1135 (76.79) |  |
| The lowest birth weight of EPIs that should receive resuscitation |  |  | <0.001 |
| 500–749g | 271 (52.72) | 199 (17.53) |  |
| 750–999g | 186 (36.19) | 362 (31.89) |  |
| ＞1000g | 57 (11.09) | 574 (50.57) |  |

*Note*. Chi-square tests were applied to categorical variables, ordinal variables were compared with Mann-Whitney U tests.

**Table S3** Attitudes towards the inappropriateness to use 28 weeks as the cutoff for providing full care.

| Variables | PR (95% CI) ^a^ | *P* |
| --- | --- | --- |
| Neonatologists *vs.* obstetricians | 1.56 (1.42-1.72) | <0.001 |
| Age | 0.99 (0.99-1.00) | 0.188 |
| Sex – female *vs.* male | 0.94 (0.85-1.04) | 0.214 |
| Han Chinese | 1.35 (1.12-1.63) | 0.002 |
| Marital status – married *vs.* others | 1.00 (0.72-1.39) | 0.993 |
| Having kids | 1.12 (0.85-1.47) | 0.437 |
| Region |  |  |
| West of China | Reference |  |
| Center of China | 1.12 (1.00-1.25) | 0.051 |
| East of China | 1.47 (1.34-1.61) | <0.001 |
| Type of hospital – others *vs.* general hospital ^b^ | 1.17 (1.08-1.27) | 0.001 |
| Hospital level – tertiary hospital *vs.* secondary hospital and below | 1.47 (1.26-1.71) | <0.001 |
| Annual number of premature infants born in the department – ≥10 *vs.* <10 | 1.41 (1.27-1.57) | <0.001 |
| Job title – chief physician *vs*. deputy chief physician | 1.19 (1.07-1.32) | 0.002 |
| Having delivered infants at <28 weeks gestation | 1.79 (1.42-2.26) | <0.001 |

^a^ PR means prevalence ratio estimated from Model 3 which adjusted for all of the variables listed in Table 1.

^b^ Others mean children hospital, maternal and child health hospital.

Abbreviation: 95% CI, 95% confidence interval.

**Table S4** Potentially influential factors of attitudes towards inappropriateness to use 28 weeks as the cutoff.

| Variables | Neonatologists | |  | Obstetricians | |
| --- | --- | --- | --- | --- | --- |
|  | PR (95% CI) | *P* |  | PR (95% CI) | *P* |
| Age | 0.98 (0.92-1.05) | 0.538 |  | 0.98 (0.95,1.00) | 0.083 |
| Sex – female *vs.* male | 1.06 (0.52-2.15) | 0.879 |  | 1.47 (0.70-3.08) | 0.312 |
| Han Chinese | 5.40 (1.94-15.03) | 0.001 |  | 1.31 (0.80-2.13) | 0.285 |
| Marital status – married *vs.* others | 5.05 (0.61-41.98) | 0.134 |  | 3.09 (0.83-11.54) | 0.094 |
| Having kids | 3.82 (0.29-50.88) | 0.311 |  | 0.45 (0.09-2.36) | 0.345 |
| Region |  |  |  |  |  |
| West of China | Reference |  |  | Reference |  |
| Center of China | 0.94 (0.44-2.01) | 0.864 |  | 1.42 (0.99-2.04) | 0.055 |
| East of China | 2.08 (0.84-5.18) | 0.114 |  | 3.06 (2.15-4.36) | <0.001 |
| Type of hospital – others *vs.* general hospital ^a^ | 1.53 (0.75-3.10) | 0.240 |  | 1.19 (0.86-1.67) | 0.298 |
| Hospital level –tertiary hospital *vs.* secondary hospital and below | 1.63 (0.73-3.63) | 0.229 |  | 1.78 (1.29-2.47) | 0.001 |
| Annual number of premature infants born in the department – ≥10 *vs.* <10 | 1.11 (0.54-2.30) | 0.775 |  | 2.25 (1.66-3.06) | <0.001 |
| Job title – chief physician *vs*. deputy chief physician | 1.38 (0.63-3.04) | 0.425 |  | 1.13 (0.81-1.59) | 0.476 |
| Having delivered infants at <28 weeks gestation | 5.26 (2.43-11.42) | <0.001 |  | 1.90 (1.23-2.95) | 0.004 |

^a^ others mean children hospital, maternal and child health hospital.

Abbreviations: PR, prevalence ratio; 95% CI, 95% confidence interval.

**Table S5** Attitudes towards the lowest gestational age of EPIs that should receive resuscitation.

| Variables | 26 *vs.* 27 weeks | |  | 25 *vs.* 27 weeks | |  | ≤24 *vs.* 27 weeks | |
| --- | --- | --- | --- | --- | --- | --- | --- | --- |
|  | OR (95% CI) ^a^ | *P* |  | OR (95% CI) | *P* |  | OR (95% CI) | *P* |
| Neonatologists *vs.* obstetricians | 1.07 (0.80-1.44) | 0.651 |  | 6.78 (4.64-9.91) | <0.001 |  | 4.49 (3.39-5.94) | <0.001 |
| Age | 0.98 (0.96-1.00) | 0.035 |  | 1.00 (0.97-1.03) | 0.797 |  | 1.00 (0.98-1.02) | 0.816 |
| Sex – female *vs.* male | 0.76 (0.50-1.15) | 0.197 |  | 0.58 (0.37-0.93) | 0.023 |  | 0.68 (0.46-1.00) | 0.051 |
| Han Chinese | 1.80 (1.25-2.60) | 0.002 |  | 1.93 (1.06-3.50) | 0.032 |  | 1.84 (1.24-2.72) | 0.002 |
| Marital status – married *vs.* others | 2.30 (0.94-5.63) | 0.069 |  | 0.41 (0.16-1.06) | 0.067 |  | 0.57 (0.28-1.18) | 0.128 |
| Having kids | 2.31 (0.94-5.68) | 0.068 |  | 0.67 (0.16-2.84) | 0.589 |  | 1.32 (0.51-3.40) | 0.568 |
| Region |  |  |  |  |  |  |  |  |
| West of China | Reference |  |  | Reference |  |  | Reference |  |
| Center of China | 1.48 (1.13-1.94) | 0.004 |  | 1.50 (0.97-2.31) | 0.069 |  | 1.20 (0.89-1.61) | 0.239 |
| East of China | 2.68 (2.02-3.55) | <0.001 |  | 3.47 (2.32-5.19) | <0.001 |  | 2.81 (2.09-3.77) | <0.001 |
| Type of hospital – others *vs.* general hospital ^b^ | 1.12 (0.87-1.44) | 0.376 |  | 1.39 (0.98-1.97) | 0.065 |  | 1.55 (1.19-2.01) | 0.001 |
| Hospital level – tertiary hospital *vs.* secondary hospital and below | 1.60 (1.24-2.06) | <0.001 |  | 2.38 (1.48-3.81) | <0.001 |  | 1.73 (1.30-2.31) | <0.001 |
| Annual number of premature infants born in the department – ≥10 *vs.* <10 | 1.39 (1.09-1.77) | 0.008 |  | 2.50 (1.74-3.59) | <0.001 |  | 2.29 (1.77-2.96) | <0.001 |
| Job title – chief physician vs. deputy chief physician | 1.17 (0.90-1.53) | 0.236 |  | 1.07 (0.71-1.59) | 0.755 |  | 1.06 (0.80-1.42) | 0.677 |
| Having delivered infants at <28 weeks gestation | 1.91 (1.40-2.61) | <0.001 |  | 3.71 (1.90-7.24) | <0.001 |  | 2.11 (1.48-3.01) | <0.001 |

^a^ OR means prevalence ratio estimated from Model 3 which adjusted for all of the variables listed in Table 1.

^b^ Others mean children hospital, maternal and child health hospital.

Abbreviations: EPIs, extreme preterm infants; 95% CI, 95% confidence interval.

**Table S6** Potentially influential factors of attitudes towards the lowest gestational age of EPIs that should receive resuscitation among neonatologists.

| Variables | 26 *vs.* 27 weeks | |  | 25 *vs.* 27 weeks | |  | ≤24 *vs.* 27 weeks | |
| --- | --- | --- | --- | --- | --- | --- | --- | --- |
|  | OR (95% CI) | *P* |  | OR (95% CI) | *P* |  | OR (95% CI) | *P* |
| Age | 0.98 (0.94-1.04) | 0.538 |  | 1.00 (0.95-1.05) | 0.931 |  | 0.99 (0.95-1.03) | 0.632 |
| Sex – female *vs.* male | 0.77 (0.44-1.36) | 0.371 |  | 0.54 (0.30-0.95) | 0.034 |  | 0.62 (0.37-1.03) | 0.064 |
| Han Chinese | 1.34 (0.60-2.99) | 0.470 |  | 2.12 (0.82-5.47) | 0.121 |  | 1.21 (0.60-2.47) | 0.592 |
| Marital status – married *vs.* others | 2.21 (0.41-11.83) | 0.354 |  | 1.00 (0.21-4.66) | 0.998 |  | 2.02 (0.48-8.54) | 0.342 |
| Having kids | 8.96 (0.93-86.71) | 0.058 |  | 2.49 (0.19-33.12) | 0.491 |  | 4.65 (0.48-44.71) | 0.183 |
| Region |  |  |  |  |  |  |  |  |
| West of China | Reference |  |  | Reference |  |  | Reference |  |
| Center of China | 1.28 (0.71-2.30) | 0.404 |  | 1.24 (0.65-2.36) | 0.520 |  | 1.06 (0.62-1.82) | 0.835 |
| East of China | 1.75 (0.92-3.35) | 0.090 |  | 3.42 (1.78-6.56) | <0.001 |  | 2.84 (1.59-5.07) | <0.001 |
| Type of hospital – others *vs.* general hospital ^a^ | 1.72 (1.01-2.92) | 0.045 |  | 1.83 (1.07-3.15) | 0.029 |  | 1.85 (1.14-3.00) | 0.012 |
| Hospital level – tertiary hospital *vs.* secondary hospital and below | 1.60 (0.87-2.95) | 0.132 |  | 3.83 (1.77-8.29) | 0.001 |  | 2.93 (1.63-5.28) | <0.001 |
| Annual number of premature infants born in the department – ≥10 *vs.* <10 | 1.26 (0.74-2.13) | 0.399 |  | 2.83 (1.65-4.87) | <0.001 |  | 4.33 (2.69-6.94) | <0.001 |
| Job title – chief physician *vs.* deputy chief physician | 1.79 (0.98-3.26) | 0.057 |  | 1.40 (0.75-2.60) | 0.295 |  | 1.53 (0.89-2.63) | 0.126 |
| Having delivered infants at <28 weeks gestation | 2.67 (1.40-5.09) | 0.003 |  | 5.49 (2.14-14.09) | <0.001 |  | 2.85 (1.55-5.23) | 0.001 |

^a^ others mean children hospital, maternal and child health hospital.

Abbreviations: EPIs, extreme preterm infants; OR, odds ratio; 95% CI, 95% confidence interval.

**Table S7** Potentially influential factors of attitudes towards the lowest gestational age of EPIs that should receive resuscitation among obstetricians.

| Variables | 26 *vs.* 27 weeks | |  | 25 *vs.* 27 weeks | |  | ≤24 *vs.* 27 weeks | |
| --- | --- | --- | --- | --- | --- | --- | --- | --- |
|  | OR (95% CI) | *P* |  | OR (95% CI) | *P* |  | OR (95% CI) | *P* |
| Age | 0.98 (0.96-1.00) | 0.051 |  | 0.98 (0.94-1.02) | 0.342 |  | 1.00 (0.98-1.03) | 0.738 |
| Sex – female *vs.* male | 0.66 (0.34-1.26) | 0.207 |  | 0.59 (0.20-1.74) | 0.342 |  | 0.57 (0.28-1.17) | 0.125 |
| Han Chinese | 1.95 (1.28-2.95) | 0.002 |  | 1.24 (0.55-2.77) | 0.606 |  | 2.40 (1.39-4.13) | 0.002 |
| Marital status – married *vs.* others | 2.66 (0.89-7.93) | 0.080 |  | 0.34 (0.10-1.18) | 0.089 |  | 0.37 (0.17-0.82) | 0.014 |
| Having kids | 1.76 (0.62-5.01) | 0.288 |  | 0.49 (0.05-4.70) | 0.536 |  | 0.99 (0.29-3.30) | 0.981 |
| Region |  |  |  |  |  |  |  |  |
| West of China | Reference |  |  | Reference |  |  | Reference |  |
| Center of China | 1.53 (1.13-2.08) | 0.007 |  | 1.77 (0.91-3.46) | 0.093 |  | 1.26 (0.87-1.84) | 0.226 |
| East of China | 2.97 (2.17-4.07) | <0.001 |  | 3.01 (1.62-5.60) | 0.001 |  | 2.75 (1.91-3.96) | <0.001 |
| Type of hospital – others *vs.* general hospital ^a^ | 0.97 (0.72-1.30) | 0.821 |  | 1.17 (0.66-2.06) | 0.588 |  | 1.48 (1.07-2.05) | 0.019 |
| Hospital level – tertiary hospital *vs.* secondary hospital and below | 1.53 (1.15-2.03) | 0.003 |  | 1.71 (0.90-3.23) | 0.100 |  | 1.57 (1.12-2.21) | 0.009 |
| Annual number of premature infants born in the department – ≥10 *vs.* <10 | 1.47 (1.12-1.94) | 0.006 |  | 3.78 (2.08-6.87) | <0.001 |  | 1.46 (1.05-2.02) | 0.025 |
| Job title – chief physician *vs.* deputy chief physician | 1.03 (0.77-1.40) | 0.829 |  | 1.06 (0.58-1.94) | 0.837 |  | 0.96 (0.67-1.38) | 0.834 |
| Having delivered infants at <28 weeks gestation | 1.74 (1.21-2.49) | 0.003 |  | 2.10 (0.80-5.48) | 0.131 |  | 1.56 (1.01-2.40) | 0.044 |

^a^ others mean children hospital, maternal and child health hospital.

Abbreviations: EPIs, extreme preterm infants; OR, odds ratio; 95% CI, 95% confidence interval.

**Table S8** Attitudes towards whether EPIs should receive resuscitation no matter how light they are.

| Variables | PR (95% CI) ^a^ | *P* |
| --- | --- | --- |
| Neonatologists *vs.* obstetricians | 1.77 (1.53-2.04) | <0.001 |
| Age | 0.99 (0.98-1.00) | 0.024 |
| Sex – female *vs.* male | 1.09 (0.90-1.30) | 0.381 |
| Han Chinese | 0.91 (0.74-1.12) | 0.384 |
| Marital status – married *vs.* others | 0.65 (0.47-0.89) | 0.008 |
| Having kids | 1.21 (0.80-1.82) | 0.366 |
| Region |  |  |
| West of China | Reference |  |
| Center of China | 0.94 (0.80-1.12) | 0.502 |
| East of China | 1.03 (0.88-1.20) | 0.749 |
| Type of hospital – others *vs.* general hospital ^b^ | 1.00 (0.87-1.15) | 0.979 |
| Hospital level – tertiary hospital *vs.* secondary hospital and below | 0.92 (0.79-1.08) | 0.328 |
| Annual number of premature infants born in the department – ≥10 *vs.* <10 | 1.09 (0.95-1.25) | 0.229 |
| Job title – chief physician *vs.* deputy chief physician | 0.91 (0.79-1.06) | 0.247 |
| Having delivered infants at <28 weeks gestation | 0.90 (0.75-1.08) | 0.259 |

^a^ PR means prevalence ratio estimated from Model 3 which adjusted for all of the variables listed in Table 1.

^b^ Others mean children hospital, maternal and child health hospital.

Abbreviations: EPIs, extreme preterm infants; 95% CI, 95% confidence interval.

**Table S9** Potentially influential factors of attitudes towards whether EPIs should receive resuscitation no matter how light they are.

| Variables | Neonatologists | |  | Obstetricians | |
| --- | --- | --- | --- | --- | --- |
|  | PR (95% CI) | *P* |  | PR (95% CI) | *P* |
| Age | 0.98 (0.96-0.99) | 0.005 |  | 1.00 (0.98-1.01) | 0.594 |
| Sex – female *vs.* male | 1.18 (0.97-1.43) | 0.107 |  | 0.72 (0.49-1.06) | 0.097 |
| Han Chinese | 0.96 (0.73-1.28) | 0.796 |  | 0.85 (0.64-1.14) | 0.281 |
| Marital status – married *vs.* others | 0.89 (0.51-1.55) | 0.685 |  | 0.57 (0.38-0.85) | 0.006 |
| Having kids | 0.64 (0.30-1.36) | 0.242 |  | 2.00 (1.26-3.16) | 0.003 |
| Region |  |  |  |  |  |
| West of China | Reference |  |  | Reference |  |
| Center of China | 0.79 (0.63-1.01) | 0.056 |  | 1.10 (0.87-1.39) | 0.435 |
| East of China | 0.94 (0.77-1.15) | 0.559 |  | 1.09 (0.86-1.37) | 0.487 |
| Type of hospital – others *vs.* general hospital ^a^ | 0.91 (0.76-1.09) | 0.301 |  | 1.08 (0.88-1.33) | 0.458 |
| Hospital level – tertiary hospital *vs.* secondary hospital and below | 0.95 (0.73-1.24) | 0.708 |  | 0.94 (0.76-1.16) | 0.585 |
| Annual number of premature infants born in the department – ≥10 *vs.* <10 | 1.31 (1.07-1.59) | 0.007 |  | 0.89 (0.73-1.10) | 0.284 |
| Job title – chief physician *vs.* deputy chief physician | 0.97 (0.79-1.20) | 0.778 |  | 0.87 (0.70-1.09) | 0.230 |
| Having delivered infants at <28 weeks gestation | 0.98 (0.73-1.31) | 0.887 |  | 0.84 (0.66-1.07) | 0.167 |

^a^ others mean children hospital, maternal and child health hospital.

Abbreviations: EPIs, extreme preterm infants; PR, prevalence ratio; 95% CI, 95% confidence interval.

**Table S10** Attitudes towards the lowest birth weight of EPIs receiving resuscitation.

| Variables | 750-999 *vs.* >1000 g | | |  | 500-749 *vs.* >1000 g | |  |
| --- | --- | --- | --- | --- | --- | --- | --- |
|  | OR (95% CI) ^a^ | *P* |  | | OR (95% CI) | *P* | |
| Neonatologists *vs.* obstetricians | 5.32 (3.62-7.82) | <0.001 |  | | 13.96 (9.33-20.89) | <0.001 | |
| Age | 0.98 (0.96-1.00) | 0.112 |  | | 0.99 (0.96-1.01) | 0.360 | |
| Sex – female *vs.* male | 1.19 (0.74-1.92) | 0.482 |  | | 1.10 (0.67-1.80) | 0.718 | |
| Han Chinese | 1.60 (1.03-2.48) | 0.037 |  | | 1.11 (0.68-1.80) | 0.672 | |
| Marital status – married *vs.* others | 3.38 (1.13-10.11) | 0.029 |  | | 1.73 (0.62-4.81) | 0.297 | |
| Having kids | 0.82 (0.26-2.62) | 0.741 |  | | 0.88 (0.27-2.92) | 0.839 | |
| Region |  |  |  | |  |  | |
| West of China | Reference |  |  | | Reference |  | |
| Center of China | 1.42 (1.04-1.95) | 0.028 |  | | 1.60 (1.10-2.32) | 0.014 | |
| East of China | 2.92 (2.12-4.03) | <0.001 |  | | 4.11 (2.85-5.91) | <0.001 | |
| Type of hospital – others *vs.* general hospital ^a^ | 1.26 (0.94-1.68) | 0.127 |  | | 1.16 (0.83-1.61) | 0.382 | |
| Hospital level – tertiary hospital *vs.* secondary hospital and below | 1.78 (1.33-2.39) | <0.001 |  | | 2.05 (1.43-2.94) | <0.001 | |
| Annual number of premature infants born in the department – ≥10 *vs.* <10 | 1.94 (1.48-2.56) | <0.001 |  | | 3.25 (2.38-4.45) | <0.001 | |
| Job title – chief physician *vs.* deputy chief physician | 1.15 (0.85-1.56) | 0.356 |  | | 1.13 (0.80-1.60) | 0.503 | |
| Having delivered infants at <28 weeks gestation | 2.24 (1.54-3.25) | <0.001 |  | | 3.63 (2.21-5.96) | <0.001 | |

^a^ OR means odds ratio estimated from Model 3 which adjusted for all of the variables listed in Table 1.

^b^ others mean children hospital, maternal and child health hospital.

Abbreviations: EPIs, extreme preterm infants; 95% CI, 95% confidence interval.

**Table S11** Potentially influential factors of attitudes towards the lowest birth weight of EPIs receiving resuscitation.

| Variables | Nneonatologists | | | | |  | Obstetricians | | | | |
| --- | --- | --- | --- | --- | --- | --- | --- | --- | --- | --- | --- |
|  | 750-999 *vs.* >1000 g | |  | 500-749 *vs.* >1000 g | |  | 750-999 *vs.* >1000 g | |  | 500-749 *vs.* >1000 g | |
|  | OR (95% CI) | *P* |  | OR (95% CI) | *P* |  | OR (95% CI) | *P* |  | OR (95% CI) | *P* |
| Age | 0.98 (0.92-1.05) | 0.538 |  | 0.97 (0.91-1.03) | 0.289 |  | 0.98 (0.95-1.00) | 0.083 |  | 1.00 (0.97-1.03) | 0.789 |
| Sex – female *vs.* male | 1.06 (0.52-2.15) | 0.879 |  | 1.09 (0.53-2.23) | 0.823 |  | 1.47 (0.70-3.08) | 0.312 |  | 0.85 (0.39-1.85) | 0.682 |
| Han Chinese | 5.40 (1.94-15.03) | 0.001 |  | 3.48 (1.33-9.12) | 0.011 |  | 1.31 (0.80-2.13) | 0.285 |  | 0.89 (0.50-1.56) | 0.674 |
| Marital status – married *vs.* others | 5.05 (0.61-41.98) | 0.134 |  | 1.99 (0.33-11.96) | 0.451 |  | 3.09 (0.83-11.54) | 0.094 |  | 1.78 (0.46-6.82) | 0.400 |
| Having kids | 3.82 (0.29-50.88) | 0.311 |  | 3.01 (0.22-40.48) | 0.407 |  | 0.45 (0.09-2.36) | 0.345 |  | 0.78 (0.14-4.24) | 0.778 |
| Region |  |  |  |  |  |  |  |  |  |  |  |
| West of China | Reference |  |  | Reference |  |  | Reference |  |  | Reference |  |
| Center of China | 0.94 (0.44-2.01) | 0.864 |  | 0.93 (0.42-2.02) | 0.846 |  | 1.42 (0.99-2.04) | 0.055 |  | 1.91 (1.21-3.02) | 0.005 |
| East of China | 2.08 (0.84-5.18) | 0.114 |  | 3.39 (1.37-8.40) | 0.008 |  | 3.06 (2.15-4.36) | <0.001 |  | 4.14 (2.68-6.38) | <0.001 |
| Type of hospital – others *vs.* general hospital ^a^ | 1.53 (0.75-3.10) | 0.240 |  | 1.34 (0.66-2.74) | 0.422 |  | 1.19 (0.86-1.67) | 0.298 |  | 1.09 (0.73-1.63) | 0.677 |
| Hospital level – tertiary hospital *vs.* secondary hospital and below | 1.63 (0.73-3.63) | 0.229 |  | 2.94 (1.25-6.91) | 0.013 |  | 1.78 (1.29-2.47) | 0.001 |  | 1.75 (1.17-2.64) | 0.007 |
| Annual number of premature infants born in | 1.11 (0.54-2.30) | 0.775 |  | 2.61 (1.27-5.35) | 0.009 |  | 2.25 (1.66-3.06) | <0.001 |  | 2.90 (2.00-4.22) | <0.001 |
| the department – ≥10 *vs.* <10 |  |  |  |  |  |  |  |  |  |  |  |
| Job title – chief physician *vs.* deputy chief physician | 1.38 (0.63-3.04) | 0.425 |  | 1.50 (0.68-3.32) | 0.320 |  | 1.13 (0.81-1.59) | 0.476 |  | 1.04 (0.69-1.57) | 0.862 |
| Having delivered infants at <28 weeks gestation | 5.26 (2.43-11.42) | <0.001 |  | 8.72 (3.73-20.42) | <0.001 |  | 1.90 (1.23-2.95) | 0.004 |  | 2.33 (1.26-4.28) | 0.007 |

Abbreviations: EPIs, extreme preterm infants; OR, odds ratio; 95% CI, 95% confidence interval.
